# Rapamycin promoted thrombosis and platelet adhesion to endothelial cells by inducing membrane remodeling

**DOI:** 10.1186/1471-2121-15-7

**Published:** 2014-02-24

**Authors:** Ping Jiang, Yong Lan, Jun Luo, Ya-Li Ren, Dong-Ge Liu, Jian-Xin Pang, Jin Liu, Jian Li, Chen Wang, Jian-Ping Cai

**Affiliations:** 1 The Key Laboratory of Geriatrics, Beijing Hospital and Beijing Institute of Geriatrics, Ministry of Health, No.1, DaHua Road, Dong Dan, Beijing 100730, P.R.China; 2 Department of Vascular Surgery, Beijing Hospital & Beijing Institute of Geriatrics, Ministry of Health, Beijing 100730, China; 3 Laboratory of Electron Microscopy, Peking University First Hospital, Beijing 100034, China; 4 Department of Pathology, Beijing Hospital & Beijing Institute of Geriatrics, Ministry of Health, Beijing 100730, China; 5 College of Lifesciences, Beijing Normal University, Beijing 100875, China; 6 Department of Respiratory Medicine, Beijing Hospital & Beijing Institute of Geriatrics, Ministry of Health, Beijing 100730, China

**Keywords:** Thrombosis, Membrane remodeling, Endothelial cell, Platelet

## Abstract

**Background:**

Recently, evidence indicated that the rapamycin-eluting stent which was used worldwide may contribute to an increased risk for thrombosis. On the contrary, other researchers found it was safe. Thus, it is necessary to clarify the effect of rapamycin on thrombosis and the corresponding mechanisms.

**Results:**

The effects of rapamycin in *vivo* were evaluated by modified deep vein thrombosis animal model. The platelets were from healthy volunteers and the platelet-endothelium (purchased from ATCC) adhesion in cultured endothelial cells was assessed. Membrane rufflings in endothelial cells were examined by confocal and electron microscope. Thrombus formation increased in rats that were injected with rapamycin. Electron microscope analysis exhibited microvilli on the rapamycin-treated endothelium in rats. Rapamycin enhanced membrane ruffling in human umbilical vein endothelial cells (HUVECs) and adhesion of platelets to HUVECs. The platelet-HUVECs adhesion was attenuated when cells were treated with cytochalacin B. Inhibition of autophagy by 3-methyladenine led to suppression of membrane ruffles in HUVECs and augmentation of platelet-endothelial adhesion.

**Conclusions:**

In conclusion, we found that endothelial membrane remodeling induced by rapamycin is crucial for the adhesion of platelets to endothelial cells and thereby for thrombosis in *vivo*, and that the endothelial membrane remodeling is autophagy dependent.

## Background

Venous thrombosis, including deep vein thrombosis (DVT) and pulmonary embolism, is a major source of morbidity and mortality worldwide [1]. Although it is accepted that the combination of so-called virchow triad, namely 1) vascular abnormalities and endothelial dysfunction, 2) hypercoagulability and 3) stasis, may play a pivotal role in the pathogenesis of venous thrombosis, the underlying mechanisms are not fully elucidated.

The pathogenesis of thrombosis involves a variety of factors among which platelet adhesion to endothelial cells is one element of importance [2,3]. The data that adhesion can occur in the mice who lack fibrinogen and VWF suggests that some pivotal mechanisms, for example, the platelet-endothelial interaction, may be involved in this process and the concomitant thrombosis [4]. We have known that the rougher the membrane surfaces are, the more the platelets adhere and the poorer the hemocompatibility is, and vice versa [5]. The above data suggested that the dynamic regulation of endothelial membrane shape may affect the process of platelet adhesion. In fact, the morphology of endothelial membrane changed with environment frequently. For example, when the transmural pressure elevated or cells were exposed to E2, projections, such as membrane ruffles, pseudopodia and microvilli, will appear on the outer surface of endothelium and stretch into the vessel lumen [6,7].

Kadandale et al. [8] identified that autophagy plays a pivotal role in blood cell cortical remodeling, with involvement in the extension of cell protrusions, such as lamellipodia and filopodia. Rapamycin is a kind of autophagy agonist, which was reported to be associated with regulation of endothelial cytoskeleton [9]. Every year, rapamycin-eluting stents are implanted in millions of patients with coronary artery disease who undergo percutaneous coronary intervention. However, evidence indicated that rapamycin-eluting stents may be associated with an increased risk for stent thrombosis when compared with bare-metal stents [4,10]. For example, Camici *et al.*[10] reported that rapamycin promoted arterial thrombosis in *vivo*. In endothelial cells, rapamycin can enhance the activity of tissue factor (TF) which is a key trigger of coagulation cascade. In addition, the effects that rapamycin inhibits tissue plasminogen activator (t-PA) and induces plasminogen activator inhibitor 1 (PAI-1) in human umbilical vein endothelial cells (HUVECs) may contribute to thrombosis associated with rapamycin-eluting stents [11]. On the contrary, Daemen *et al.* and others [12,13] found that rapamycin-eluting stents were safe and effective compared with bare-metal ones. Thus, it is necessary to elucidate the effect of rapamycin on thrombosis.

In this study, we found that rapamycin (500 ng/kg) promoted formation of microvilli-like structure in endothelium and thrombotic occlusion in the modified DVT rat model. HUVECs treated with rapamycin demonstrated that both dorsal ruffling and platelets-endothelial adhesion were promoted. Suppressing dorsal ruffling by cytochalasin B led to inhibited platelet-endothelial adhesion. Further analysis suggested that rapamycin-mediated autophagy activation may contribute to the formation of the dorsal ruffling in endothelial cells.

## Results

### Rapamycin promoted thrombus formation in vivo

**Figure 1:**
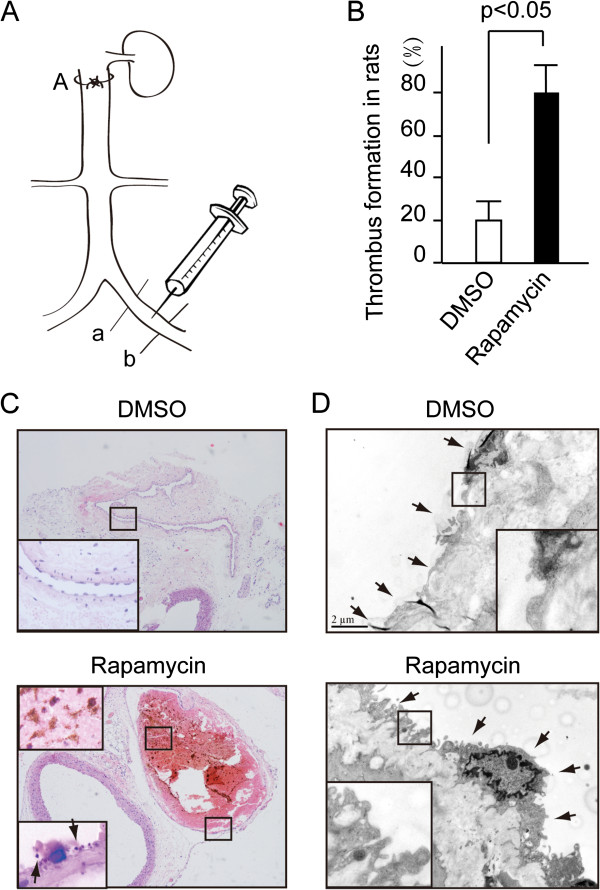
**Rapamycin promoted thrombosis in rats. (A)** The diagram of the DVT rat model illustrated that the iliac vein which was tied at site A was injected with rapamycin or DMSO and then tied tightly at the sites of a and b. **(B)** The ratio of thrombosis in rapamycin group (80%) is far more than that in DMSO (20%) group. n = 10 in each group. **(C)** HE staining exhibited thrombosis in the venous lumen at the time of the 40th minute. Enlarged squares indicate the platelet-derived clot appeared in the thrombus. **(D)** Transmission electron microscope analysis exhibited microvilli-like structure on endothelial of rapamycin treated rats compared with the smooth endothelial surface in the DMSO treated rats (TEM × 10, 000).

To clarify the effect of rapamycin on thrombosis, modified DVT rat model was adopted in this study (Figure 1A). Rapamycin (500 ng/kg) or DMSO (control) was injected into iliac vein as shown in Figure 1A when the IVC was tied with silk suture. The vein is darkred before the formation of thrombotic occlusion. After the occlusion developed the veins became black and purple. Thrombosis was confirmed by histological observation. The appearance of an occlusive thrombus was clearly visible through the microscope in the lumen of the vessels. The time-course of effects of rapamycin on thrombotic occlusion was shown in Additional file 1: Figure S1. At the time of the 40th minute, thrombotic occlusion developed in 20% of the DMSO-injected rats and 80% of the rapamycin rats (Figure 1B, *P* < 0.05). Hematoxylin and eosin staining of the venous cross section showed that the thrombus occluded in the enlarged venous lumen of rapamycin-treated rats (Figure 1C). A lot of platelet-derived clots were found to speckle in the red thrombus or attach to the vascular intima (Figure 1C, the enlarged image in the lower panel). Of note, large amount of microvilli-like structures (Figure 1D, indicated by arrow) appeared on the surface of the endothelial membrane in rapamycin-treated rats. By contrast, the endothelial membrane treated with DMSO was smooth as shown by the ultrastructure of the venous endothelium (Figure 1D). Microvilli-like structure was not obviously observed in the 20% of DMSO-injected rats with positive thrombotic occlusion and rats that were negative for thrombotic occlusions with 20% of rapamycin injection.

### Rapamycin enhanced platelet adhesion to endothelium

**Figure 2:**
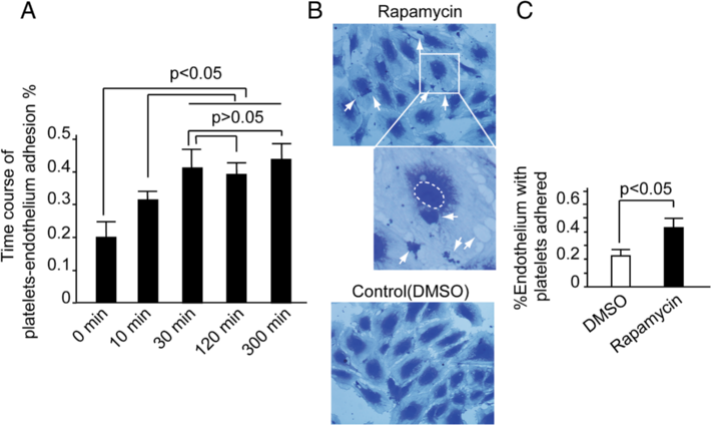
**Rapamycin induced platelets-endothelial adhesion.** HUVECs were cultured on the gelatin-coated coverslips. Freshly isolated platelets were added to the coverslip and incubated for the indicated time. Cells were fixed and stained with crystal violet. The coverslips were mounted and imaged by microscopy. **(A)** The time course of platelet-endothelial adhesion exhibited that the platelets-endothelial adhesion reached its plateau at the 30th minute. Columns, means of three independent experiments; bars, SD. **(B)** The upper and middle panel indicated that platelets were seen adhering on the HUVECs. **(C)** HUVECs with platelets adhered were calculated and significant difference existed between the two groups (*P* < 0.05). Columns, means of three independent experiments; bars, SD.

Recently, Iba *et al.* reported that adhesion of leukocytes to endothelium was the first event initiating thrombus formation [11-14]. We have known that rough membranes can enhance platelet adhesion. Thus we want to know whether rapamycin can promote platelet-endothelial adhesion. First, the time course of platelet-endothelium adhesion was evaluated as shown in Figure 2A. The amount of endothelium with platelet adhered increases with time. The maximum-adhesion point appears at the 30th minute and reaches its plateau. DMSO-treated platelets-endothelium adhesion was also assessed (Additional file 1: Figure S1A). Thus in the following experiments we chose the 30th minute as the time point. Then HUVECs were incubated with platelets, together with rapamycin (10 nM) or DMSO treatment for 30 minutes. The platelet-endothelial adhesion was evaluated. Results showed that rapamycin enhanced adhesion activity between HUVECs and platelets. The quantity of HUVECs with platelets adhered increased by 115% in rapamycin treated group compared with that in DMSO (Figure 2B and 2C, *P* < 0.05).

### Rapamycin induced membrane remodeling in endothelium

**Figure 3:**
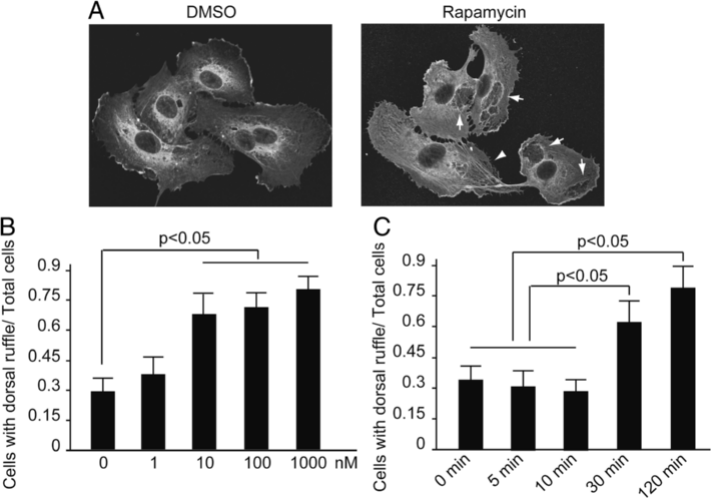
**Rapamycin promoted membrane remodeling in HUVECs. (A)** HUVECs were incubated with rapamycin (10 nM) for 30 minutes. Cells were fixed and detected by immunofluorescence with an anti-β-actin antibody. The representative images showed the formation of dorsal ruffle. **(B)** HUVECs were treated with the indicated concentration of rapamycin for 30 minutes. Cells were fixed and detected by immunofluorescence with an anti-β-actin antibody. The ratio of cells with dorsal ruffle to total cells was evaluated. Notice that rapamycin at the concentration of 10 to 1000 nM can lead to dramatically enhanced dorsal ruffle formation. Columns, means of three independent experiments; bars, SD. **(C)** HUVECs were incubated with rapamycin (10 nM) for the indicated time. Cells were fixed and detected by immunofluorescence with an anti-β-actin antibody. The ratio of cells with dorsal ruffle to total cells was evaluated. Means ± SD are given and were obtained from 3 independent experiments.

As we have found, rapamycin induced microvilli-like structure in the endothelial cells of mice. To have an insight into the effect of rapamycin on the cultured endothelial cells, HUVECs treated with rapamycin were investigated by confocal microscopy. When HMVECs were exposed to 10 nM of rapamycin, peripheral and dorsal ruffles were found to arise from peripheral and central of the surface (Figure 3A, lower panel) by detection of F-actin organization. By comparison, central ruffles were observed little in the control (Figure 3A, upper panel). Then HUVECs treated with different concentration of rapamycin were examined at different time points by the way of immunofluorescence detection to quantify membrane remodeling induced by rapamycin.

As shown in Figure 3B and 3C, rapamycin at the concentration of 10 to 1000 nM can lead to dramatically enhanced dorsal ruffle formation. And the formation of prominent dorsal ruffles peaks at the 30th minute after rapamycin stimulation.

### The membrane remodeling is necessary for platelet-endothelium adhesion

**Figure 4:**
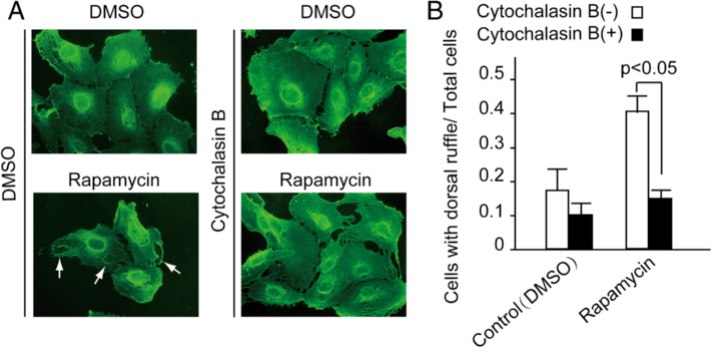
**The membrane remodeling is essential for platelet-endothelial adhesion.** HUVECs were treated with the indicated reagents. Cells were fixed and detected by immunofluorescence with an anti-β-actin antibody. **(A)** The dorsal ruffle was indicated by arrows. **(B)** The ratio of cells with dorsal ruffle to total cells was evaluated. The data showed that Cytochalasin B attenuated platelets-endothelial adhesion which was induced by rapamycin. Means ± SD are given and were obtained from 3 independent experiments.

To show where platelet is going to interact with endothelium, we added the platelets to the cultured endothelium. As was shown in Additional file 1: Figure S1C, platelets are apt to associate with endothelial cells at the site of ruffles and filopodialike protrusions of the latter. Then cytochalasin B, a drug that can bind to actin filaments and block polymerization and elongation of actin, was added to inhibit the formation of dorsal ruffle in order to explore the effect of the ruffles on platelet-endothelial adhesion [15]. As shown in Figure 4A, HUVECs exhibited reduced dorsal ruffles when incubated with 30 μM cytochalasin B for 20 minutes even under the stimulation of rapamycin. Platelet adhesion was inhibited after the HUVECs were treated with cytochalasin B (Figure 4B). Compared with the control (DMSO treatment), the platelet-endothelium adhesion increased by 120% and 50% respectively, in rapamycin incubated group and rapamycin + cytochalasin B group. These data indicated that the dorsal ruffle formation of HUVECs is essential for the adhesion activity between platelets and endothelium.

### The membrane remodeling was induced by autophagy

**Figure 5:**
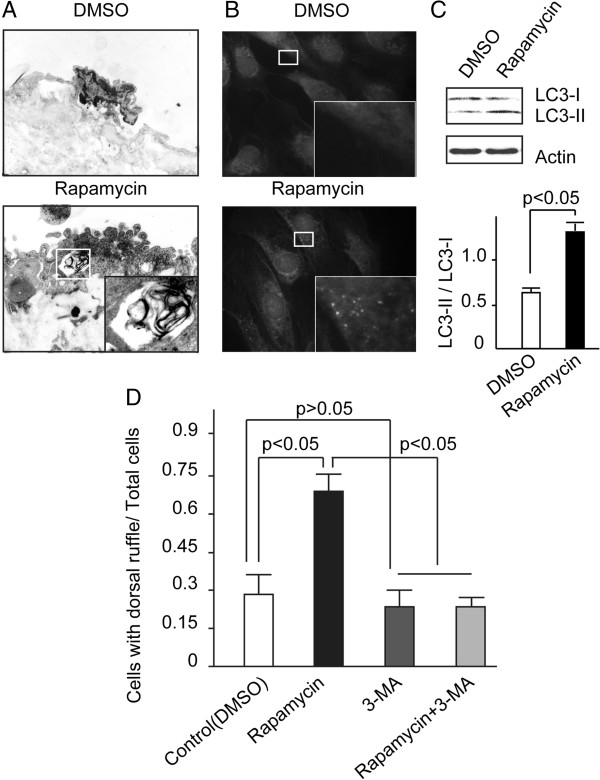
**The membrane remodeling was induced by autophagy. (A)** Transmission electron microscope analysis exhibited the induction of autophagy in the venous endothelium. Enlarged square showed the autophagosome (TEM × 10, 000). **(B, C)** HUVECs were treated with the indicated reagents, followed by fixation and detection by immunofluorescence (B) and western blot **(C)** with anti-LC3-II antibody. The representative images indicated the dot-like structure of autophagosomes in the cells **(B)** and the expression of LC3-II in cells **(C)**. Means ± SD are given and were obtained from 4 independent experiments. **(D)**, HUVECs were treated with the indicated reagents, and the formation of dorsal ruffle was analyzed. The data exhibited that rapamycin induced dorsal ruffling. While 3-MA can suppress the formation of dorsal ruffle which was promoted by rapamycin. Means ± SD are given and were obtained from 3 independent experiments.

Recently, it was reported that autophagy was essential for the cortical remodeling of *Drosophila* blood cells (hemocytes) and mouse macrophages. In this study, autophagic activity of HUVECs was assessed by electron microscopy and immunohistochemistry to investigate the effect of rapamycin, one of the autophagy agonists. As expected, autophagosomes, which were characterized by double-membraned vesicles that contain cytosol or morphologically intact cytoplasmic organelles, could be observed in the endothelium of rapamycin treated rats (Figure 5A, enlarged square in lower panel). In contrast, autophagic activity did not increase in the DMSO treated endothelium. Immunohistochemical analysis showed that the intensity of LC3-II, which is one of the markers indicating autophagic activity, was stronger in endothelial cells treated with rapamycin than DMSO (data not shown). Next, autophagic activity induced by rapamycin was examined in the cultured HUVECs. As could be seen in Figure 5B, rapamycin incubation leads to aggregated fluorescent dots in the HUVECs. In parallel, the ratio of LC3-II/LC3-I, which was used as the marker of autophagy [16], of the rapamycin group is higher than the DMSO group (Figure 5C). To explore the importance of rapamycin-mediating autophagic activation to endothelial dorsal ruffling, we chose 3-MA, which can block autophagosome formation via inhibition of type III phosphatidylinositol 3-kinases, as the autophagy inhibitor. 3-MA (5 nM) inhibits dorsal rufflings formation induced by rapamycin by 67% as shown in Figure 5D. In addition, starvation, which is a classic autophagy inducing factor, enhanced the formation of membrane ruffle in the HUVECs (data not shown). These data suggested that the autophagic activity induced by rapamycin is responsible for the formation of dorsal ruffle in HUVECs.

## Methods

### Reagents

Anti-LC3-II antibody and anti-mouse IgG-FITC antibody were obtained from Medical & Biological Laboratoris CO., LTD (#025, #107). Rapamycin, 3-methyladenine, and Cytochalasin B were purchased from Sigma (#R878, #M9281, C6762). Rhodamine labeled Goat anti-Rabbit IgG antibody was from Kirkegaard & Perry Laboratories, Inc. (#110144).

### Cell culture and preparation of washed human platelet suspensions

HUVECs were purchased from ATCC and cultured in Endothelial cell medium (containing 10% FBS, 1% ECGS, and 1% penicillin/streptomycin solution) under a 5% CO_2_/95% air atmosphere in a humidified incubator at 37°C. Cells were used between passages 3 and 10.

Venous blood was drawn from healthy volunteers. Platelet-rich plasma (PRP) was prepared by centrifugation at 500 g for 15 minutes at room temperature, and then platelets were pelleted by centrifugation of the PRP for 6 minutes at 2000 g and washed in HEPES buffer warmed to 37°C (0.137 M NaCl, 2.68 mM KCl, 1 mM MgCl_2_, 1 mM CaCl_2_, 5 mM HEPES, and 0.1% glucose, pH 6.8).

The work was approved by the Ethics Committee of the National Center for Clinical Laboratories, and adhered to the tenets of the Declaration of Helsinki. Written informed consents were obtained from the donors.

### Platelet adhesion assay

The platelet adhesion assay was performed as previously described [17] by some modification. In brief, HUVECs were seeded on a gelatin coated coverslips at a concentration of 5 × 10^5^/ml and grown to confluence. Washed platelets (50 × 10^6^/μl) were added to the coverslips and incubated for 20 minutes under vibrating at 37°C. Non-adhered platelets were removed by washing with PBS. Cells were fixed in 3.7% paraformaldehyde for 15 minutes at room temperature and then stained with crystal violet for 10 minutes at 37°C. Cells on the coverslips were mounted and imaged by microscopy. The number of cells with platelets adhered was evaluated in triplicate per × 40 magnification field.

### Animal model

Male SD rats (180 ± 30 g) were maintained under clean conventional conditions (24 ± 2°C, 12 h light/dark cycle) and were allowed free access to water and food. The DVT model was made as previously described [18]. In brief, the inferior vena cava (IVC) was exposed via a midline incision in the abdomen followed by retraction of intestines. The IVC was carefully dissected away from the aorta for a distance of 2–3 mm immediately inferior to the renal arteries. Upon injecting rapamycin (500 ng/kg) or DMSO (control) into iliac vein, the IVC was tied with a silk suture for 40 minutes. This procedure thus created approximately a 100% cross-sectional surface stenosis and a reduction of flow upstream of the suture. Then the IVC was removed and fixed in 3.7% paraformaldehyde for subsequent Hematoxylin and eosin staining and transmission electron microscopy (TEM) analysis.

All animal procedures were performed in accordance with the National Institutes of Health Animal Care and Use Guidelines. All animal protocols were approved by the Animal Ethics Committee at the Beijing Institute of Geriatrics.

### Immunofluorescence staining

Cells cultured on gelatin-coated coverslips were washed in PBS and fixed in 3.7% formaldehyde for 15 minutes at room temperature. After blocked with 10% goat serum for 15 minutes, the coverslips were incubated with primary antibody for 60 minutes and fluorescently labeled secondary antibodies for 45 minutes at 37°C successively, and then washed extensively and mounted. Cells were viewed by use of a Bio-Rad MRC 600 laser scanning confocal microscope.

### Electron microscopy

Cells or tissues were fixed in ice-cold 1.0% glutaraldehyde in 0.1 mol/L PBS and preserved at 4°C for further processing. When processing resumed, the cells or tissues were postfixed in 1% osmium tetroxide in the same buffer, dehydrated in graded alcohols, embedded in Epon 812, sectioned with ultramicrotome (Leica, Germany), and then stained with uranyl acetate and lead citrate. The sections were examined with a transmission electron microscope (JEOL-1230, Japan).

### Statistical analysis

All data were analyzed with SPSS statistics software (Version 13.0, Chicago, IL, USA). Results were represented as mean ± standard deviation. Statistical analysis was performed using the one-way analysis of variance (ANOVA) or independent *t*-tests. A *P*-value less than 0.05 was considered statistically significant.

## Discussion

By intravital microscopic examination, Iba *et al.* found that in the venous occlusion rat model adhesion of leukocytes to endothelium was the first event after clamping followed by minute leukocyte-platelet clusters. These leukocyte-platelet aggregates move from venule to vein and finally formed a venous thrombus [14]. Thus it is important to identify what kind of factors affect the adhesion between endothelium and leukocytes. One of the possibilities, we speculated, is the remodeling of cell membrane. Emerging evidence showed that the disturbance of cellular membrane plays a pivotal role in thrombosis. For example, neutrophils interact with platelets through membrane tethers, which procedure, the authors believe, will be important in the process of inflammation and thrombosis [19]. Data indicated that platelet filopodia formation mediated by Cdc42 was required for platelet aggregation [20]. And the development of membrane tethers was essential for platelets aggregation and concomitant thrombosis [21,22]. Another sample is that tumor vessels often exhibit ‘endothelial abnormalization', characterized by a pseudostratified, hyperactive endothelium with filopodialike protrusions. In parallel, tumor vessels often show signs of thrombotic occlusion [23]. Perhaps the rough surface of endothelium is one of the reason for thrombosis.

Theoretically, the development of membrane ruffle/tethers will increase the area for platelets to contact with endothelium so that platelets have more opportunity to interact with endothelial cells. In our study, platelets from the rapamycin-treated thrombolic rat showed more filopodia (data not shown). In addition, membrane ruffles in HUVECs and microvilli-like structures in the venous endothelium increased dramatically under the condition of rapamycin incubation. It has been found that endothelial microvilli are necessary for lymphocyte-endothelium interaction [24], thus we speculate that the enhanced membrane ruffles, microvilli (or filopodia) in both endothelium and platelets, will strengthen the interaction between them and lead to thrombosis.

Hypoxia and nutrient shortage, which are apt to occur under the condition of stasis (eg. bed rest >3 days, air travel >8 hours), can induce marked upregulation of autophagy in hemocytes and endothelial cells [25,26]. The activated autophagy affected the rates of thrombosis [27,28] of which the detailed mechanisms are not clear. Recently, it was identified that autophagy was essential for cytoskeleton remodeling [8,9]. Researchers found that hemocyte-targeted RNAi depletion of autophagy-related genes (Atg4, Atg6, Atg7, Atg8a and Atg9) abolished the ability of hemocytes to spread and extend F-actin protrusions. Mammalian macrophages disrupted for autophagy remained predominantly circular in shape. In addition, live cell imaging suggests that autophagy might contribute to the cell protrusion attachment or extension [8]. More and more evidence suggested that rapamycin promote thrombosis (the mechanisms include rapamycin reducing t-PA expression and inducing PAI-1/TF expression [10,11,27]). These conclusions were confirmed in our experiment. In addition, we believe that membrane remodeling induced by rapamycin is one of the responsible mechanisms.

## Conclusions

In conclusion, we found that rapamycin stimulation induced membrane remodeling in endothelial cells. And the platelet-endothelial adhesion was enhanced in parallel. Further exploration suggested that autophagy induced by rapamycin promoted membrane remodeling, platelet-endothelial adhesion, and the concomitant thrombosis.

## Abbreviations

IVC: Inferior vena cava; DVT: Deep vein thrombosis; HUVECs: Human umbilical vein endothelial cells; 3-MA: 3-methyladenine.

## Competing interests

The authors declare that they have no competing interests.

## Authors’ contributions

PJ and JLuo carried out the cellular studies, PJi and YL drafted the manuscript. YL participated in drawing blood from the healthy volunteers. Y-LR performed the electronic microscopic analysis. D-GL and J-XP carried out the immunoassays. JLiu performed the immunofluorescence analysis. JLi, CW and J-PC conceived of the study, and participated in its design and coordination and helped to draft the manuscript. All authors read and approved the final manuscript.

## Supplementary Material

Additional file 1: Figure S1 (A) The time-course of effects of rapamycin on thrombotic occlusion in rats. Data exhibited that rapamycin promoted thrombotic occlusion with time. n = 5 in each group. (B) HUVECs were cultured on the gelatin-coated coverslips. Freshly isolated platelets, together with DMSO, were added to the coverslip and incubated for 30 minutes. Cells were fixed and stained with crystal violet. The coverslips were mounted and imaged by microscopy. The time course of platelet-endothelial adhesion was evaluated. Columns, means of three independent experiments; bars, SD. (C). Platelets attached on the endothelium at the site of ruffles (upper panel) and filopodialike protrusions (lower panel). HUVECs were cultured on the coverslips and then PKH26 labeled platelets were added into the medium. Cells were washed, fixed and stained with (upper panel) or without (lower panel) anti-β-actin antibody and the second fluorescent antibody. Then cells were analyzed with microscopy (lower panel) or immunofluorescence microscopy (upper panel). Click here for file
