# Supplementary figures and images for: Rapamycin promoted thrombosis and platelet adhesion to endothelial cells by inducing membrane remodeling
Source: BMC Cell Biol. 2014 Feb 24;15:7. doi: 10.1186/1471-2121-15-7 (PMC3936831; doi:10.1186/1471-2121-15-7)

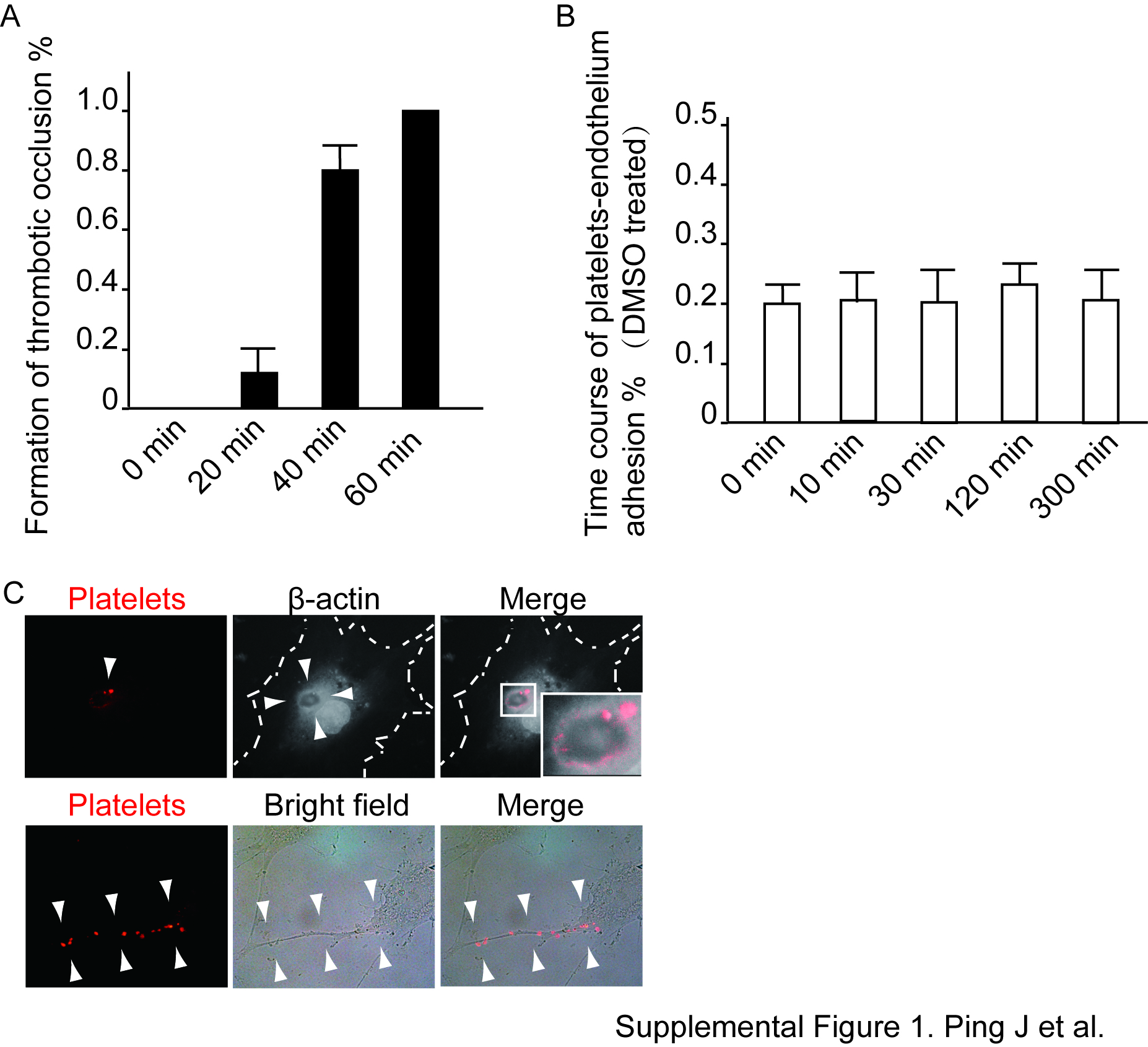

Supplement: Additional file 1: Figure S1 — (A) The time-course of effects of rapamycin on thrombotic occlusion in rats. Data exhibited that rapamycin promoted thrombotic occlusion with time. n = 5 in each group. (B) HUVECs were cultured on the gelatin-coated coverslips. Freshly isolated platelets, together with DMSO, were added to the coverslip and incubated for 30 minutes. Cells were fixed and stained with crystal violet. The coverslips were mounted and imaged by microscopy. The time course of platelet-endothelial adhesion was evaluated. Columns, means of three independent experiments; bars, SD. (C). Platelets attached on the endothelium at the site of ruffles (upper panel) and filopodialike protrusions (lower panel). HUVECs were cultured on the coverslips and then PKH26 labeled platelets were added into the medium. Cells were washed, fixed and stained with (upper panel) or without (lower panel) anti-β-actin antibody and the second fluorescent antibody. Then cells were analyzed with microscopy (lower panel) or immunofluorescence microscopy (upper panel). [file 1471-2121-15-7-S1.jpeg]
